# [(CH_3_)_2_NH_2_]_2_PdBr_4_, a layered hybrid halide perovskite semiconductor with improved optical and electrical properties†

**DOI:** 10.1039/d3ra04085b

**Published:** 2023-08-03

**Authors:** Kawther Trabelsi, Nidhal Drissi, Fadhel Hajlaoui, Mustapha Zighrioui, Abdallah Rhaiem, Nathalie Audebrand, Thierry Roisnel, Karoui Karim

**Affiliations:** a Laboratoire des Caractérisations Spectroscopiques et Optique des Matériaux, Faculté des Sciences de Sfax, Université de Sfax B.P. 1171 3000 Sfax Tunisia karouikarim36@yahoo.com + 0021625648756; b Department of Physics, Faculty of Science, King Khalid University P.O. Box 9004 Abha 61413 Saudi Arabia; c Laboratoire Physico-chimie de l’Etat Solide, Département de Chimie, Faculté des Sciences de Sfax, Université de Sfax B.P. 1171 3000 Sfax Tunisia; d GREMAN UMR, 7347-CNRS, CEA, INSACVL, Université de Tours Blois France; e Univ Rennes, CNRS, INSA Rennes, ISCR (Institut des Sciences Chimiques de Rennes) - UMR 6226 F-35000 Rennes France

## Abstract

Inspired by the success of three-dimensional hybrid perovskites (CH_3_NH_3_)PbX_3_ (X = Cl, Br, I), two-dimensional (2D) organic–inorganic hybrid metal halides have drawn immense attention due to their highly tunable physical properties. Moreover, although 3D hybrid perovskite materials have been reported, the development of new organic–inorganic hybrid semiconductors is still an area in urgent need of investigation. Here, we used the dimethylammonium cation to construct a palladium-based halide perovskite material [(CH_3_)_2_NH_2_]_2_PdBr_4_ with a 2D layered structure. This layered perovskite undergoes one endothermic peak at 415 K corresponding to melting of the organic molecule. The thermal stability of the compound is up to about 500 K. The activation energy and conduction mechanisms are discussed, and the optical study shows a gap energy equal to 2.5 eV. The electrical AC conductivity is in the order of 10^−4^ Ω^−1^ cm^−1^, which confirms the semiconductor character of this material and indicates its importance in the optoelectronic domain.

## Abstract 2

Inspired by the success of three-dimensional hybrid perovskites (CH_3_NH_3_)PbX_3_ (X = Cl, Br, I), two-dimensional (2D) organic–inorganic hybrid metal halides have drawn immense attention due to their highly tunable physical properties.

## Graphical abstract

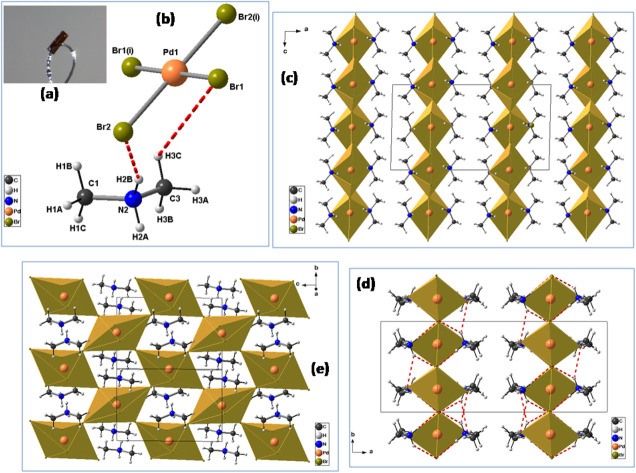

## 1. Introduction

Research on organic–inorganic metal halides is crucially important, of which the goal is to mitigate the negative environmental impacts from traditional resources and to enable ease of production. Until today, organic–inorganic metal halide perovskites have attracted much attention due to their unique properties and remarkable device performance. Compared to 3D hybrid halide perovskites, the 2D perovskite-like structures have very similar properties to lead perovskites, as well as promising photovoltaic performance.^1^ These hybrid materials have gained a lot of attention owing to their interesting physical properties, including a large optical absorption window with high absorption,^2^ good charge carrier mobility characterized by a longer lifetime,^3,4^ large diffusion lengths,^5^ and an easy manufacturing process using simpler and less expensive techniques. Furthermore, the presence of organic molecules creates a natural quantum well in the 2D perovskite structure, where the organic layer serves as a barrier and the inorganic layer functions as a well.^6^ The compound (CH_3_NH_3_)_2_PbI_3_ features a good band gap (1.55 eV) as well as a robust near-infrared and photoelectric response.^7^ However, the Pb metal transition has a high cost. Many researchers are interested in perovskite materials with reduced gap energy and lower lead content, mixed with other metals to minimize the environmental damage due to lead toxicity.^8^ Moreover, we are interested in the [NH_2_(CH_3_)_2_]^+^ cation because it is richer than [N(CH_3_)_4_]^+^ and [NH(CH_3_)_3_]^+^ in N–H bonds, which provide structural stability through the formation of hydrogen bonds.^9^ For instance, [(CH_3_)_2_NH_2_]PbX_3_ (X = Cl^−^, Br^−^, I^−^), which is in the ABX_3_ perovskite family, with a first-order phase transition for the chloride, bromide and iodide at 320 K and 250 K, respectively, associated with the motion of the organic cations.^10,11^ In the crystals, [(CH_3_)_2_NH_2_]^+^ cations and [PbX_6_]^4−^ anion chains are connected *via* hydrogen bond interactions. Both have the potential to reorient with the change of temperature. The three hybrids are semiconductors with band gap values of 3.5 eV (X: Cl^−^), 3.0 eV (X: Br^−^) and 2.59 eV (X: I^−^).

Encouraged by these pioneering works, we focused on a combination of dimethylammonium cations [(CH_3_)_2_NH_2_]^+^, the d^8^ cation Pd^2+^ and halide anion Br^−^, which leads to the synthesis of a hybrid palladium halide, [(CH_3_)_2_NH_2_]_2_PdBr_4_. The 2D hybrid perovskite material shows a strong correlation between structure and the optical and electrical properties. In this context, the 2D Pd-based material [(CH_3_)_2_NH_2_]_2_PdBr_4_ was successfully synthesized to yield a new lead-free material with reduced gap energy and high conductivity that exhibits semiconductor properties and has potential in the application area of photovoltaic cells. This accurate molecular design strategy gives research a direction to further explore Pd-based hybrid halide perovskites with promising semiconductor properties.

## 2. Experimental

### 2.1. Experimental reagents

PdBr_2_ (99%, Sigma-Aldrich), (CH_3_)_2_NH·HCl (99%, Sigma-Aldrich), HBr (ACS reagent, 48%) and ethanol were purchased and used without any further purification.

### 2.2. Synthesis details

**Fig. 1:**
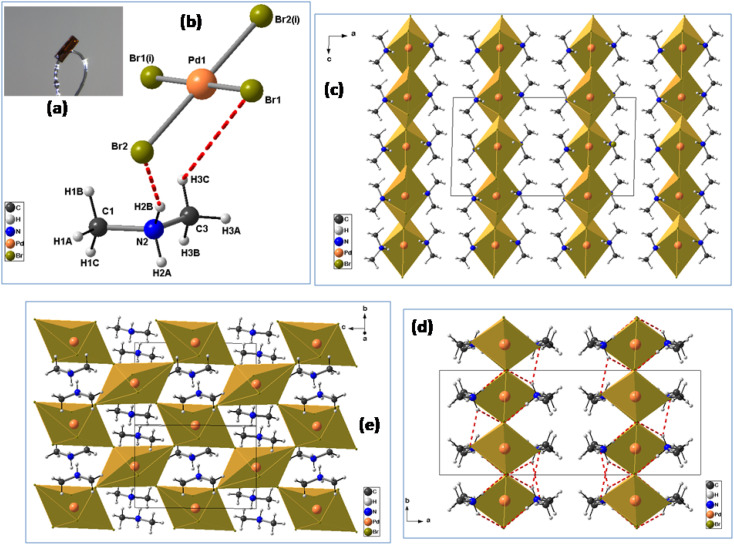
The structural assembly process of compound [(CH_3_)_2_NH_2_]_2_PdBr_4_: (a) view of the [(CH_3_)_2_NH_2_]_2_PdBr_4_ single crystal showing a lamellar nature; (b) the asymmetric unit of [(CH_3_)_2_NH_2_]_2_PdBr_4_; (c) packing diagram of [(CH_3_)_2_NH_2_]_2_PdBr_4_ along the *b*-direction; (d) packing structure of the compound along the *c*-axis; (e) extended network structure connected by corner-shared octahedra (view of 2D [PdBr_4_]^2−^ reticulated framework). Hydrogen bonds are shown with red dashed lines. Symmetry codes: (i) −*x* + 3/2, −*y* + 3/2, −*z* + 1.

The crystals were synthesized using the slow evaporation method. Firstly, a mixture of (CH_3_)_2_NH·HCl (2 mmol), PdBr_2_ (1 mmol), 10 mL of absolute ethanol and 1 mL of concentrated HBr was dissolved in water and stirred for half an hour, then heated at 50 °C and allowed to cool down naturally to room temperature. Orange plate-like single crystals of [(CH_3_)_2_NH_2_]_2_PdBr_4_ were obtained after several days by slow evaporation at room temperature (61% yield based on Pd) (Fig. 1(a)). The dimensions of the crystals are a few millimeters (Fig. S1†).

### 2.3. Single-crystal X-ray diffraction

A suitable orange plate crystal was selected for a single-crystal X-ray diffraction. The crystal was mounted on a goniometer head of a D8 Venture diffractometer with a (CMOS) PHOTON 100 detector and Mo-Kα radiation (*λ* = 0.71073 Å, multilayer monochromator) at *T* = 150 K. Crystal structure has been determined to be in monoclinic symmetry and *C*2/*c* centrosymmetric space group. The structure was solved by dual-space algorithm using the SHELXT program,^12^ and then refined with full-matrix least-squares method based on *F*^2^ with the program SHELXL-97.^13^ Anisotropic atomic displacement parameters were applied to all non-hydrogen atoms, and the H atoms were included in their calculated positions and treated as riding on their parent atom with constrained thermal parameters. The crystallographic data and structure refinement information are given in Table 1, while the Tables 2 and 3 present the selected bond distances and angles. A CIF file with atomic coordinates and anisotropic displacement parameters is available with CCDC number 2132932.

**Table 1:** Crystallographic data and structure refinement parameters for [(CH_3_)_2_NH_2_]_2_PdBr_4_

Empirical formula	C_4_H_16_N_2_PdBr_4_
Formula weight (g mol^−1^)	518.23
Temperature (K)	150
Crystal system	Monoclinic
Space group	*C*2/*c*
*a* (Å)	18.182 (2)
*b* (Å)	7.2741 (7)
*c* (Å)	9.8478 (10)
*β*	91.340 (4)
*V* (Å^3^)	1302.1 (2)
*Z*	4
*λ* (MoKα) (Å)	0.71073
*ρ*_cal_ (g cm^−3^)	2.644
Absorption correction	Multi-scan
*μ* (mm^−1^)	13.66
Crystal size (mm^3^)	0.37 × 0.12 × 0.03
Crystal color/shape	Orange/plate
*hkl* range	−23 ≤ *h* ≤ 23; 0 ≤ *k* ≤ 9; 0 ≤ *l* ≤ 12
*θ* range for data collection (°)	3.016 to 27.526
Refinement method	Full-matrix least-squares on F2
No. of collected reflections	3364
No. of independent reflections	1503
Observed reflections/restrains/parameters/refined parameters	1360/0/52
*R*_int_	0.0631
*F*(000)	960
GOF on *F*^2^	1.12
Transmission factors	*T*_min_ = 0.417, *T*_max_ = 0.620
*R* indices	*R*_1_ = 0.055, *wR*_2_ = 0.208
Min/Max (*ρ*/*e* Å^−3^)	−1.52/1.86
CCDC no.	2132932

**Table 2:** Selected bond distances (Å) and angles (°) for [(CH_3_)_2_NH_2_]_2_PdBr_4_^a^

Pd1–Br1	2.4497 (11)	C1–N2–C3	115.9 (10)
Pd1–Br1^(i)^	2.4497 (11)		
Pd1–Br2	2.4407 (10)		
Pd1–Br2^(i)^	2.4407 (10)		
Br1^(i)^–Pd1–Br1	180.0		
Br1^(i)^–Pd1–Br2^(i)^	90.13 (4)		
Br1–Pd1–Br2^(i)^	89.87 (4)		
Br1^(i)^–Pd1–Br2	89.87 (4)		
Br1–Pd1–Br2	90.13 (4)		
Br2^(i)^–Pd1–Br2	180.0		
N2–C1	1.457 (14)		
N2–C3	1.463 (16)		

a Symmetry codes: (i) −*x* + 3/2, −*y* + 3/2, −*z* + 1.

**Table 3:** Hydrogen-bonding geometry (Å, °) for [(CH_3_)_2_NH_2_]_2_PdBr_4_^a^

D–H⋯A	D–H	H⋯A	D⋯A	D–H⋯A
C1–H1A⋯Br1^(ii)^	0.98	2.93	3.798 (11)	149
C1–H1A⋯Br2^(iii)^	0.98	3.08	3.893 (12)	141
N2–H2A⋯Br1^(iv)^	0.91	2.63	3.417 (10)	145
N2–H2A⋯Br2^(v)^	0.91	2.86	3.489 (9)	128
N2–H2B⋯Br2	0.91	2.48	3.341 (9)	158
C3–H3A⋯Br1^(vi)^	0.98	2.99	3.729 (11)	134
C3–H3A⋯Br2 ^(v)^	0.98	3.01	3.622 (13)	122
C3–H3A⋯Br2^(vi)^	0.98	3.15	3.989 (12)	145
C3–H3C⋯Br1	0.98	2.99	3.767 (13)	137

a Symmetry codes: (ii) *x*, −*y* + 1, *z* + 1/2; (iii) −*x* + 3/2, *y* − 1/2, −*z* + 3/2; (iv) *x*, *y* − 1, *z*; (v) −*x* + 3/2, −*y* + 1/2, −*z* + 1; (vi) *x*, −*y* + 1, *z* − 1/2.

### 2.4. Powder X-ray diffraction

The powder X-ray diffraction (PXRD) data of the ground crystals were collected at room temperature on a PANalyticalX'Pert MPD diffractometer with Cu Kα_1,2_ radiation and equipped with a X'Celerator detector in the range of 4° < 2*θ* < 75°. The experimental X-ray powder diffraction pattern is in very good agreement with the calculated pattern generated from the single-crystal structure (in *Cmca*), evidencing that the batch contains the title compound as a unique phase. Indexing of the powder pattern was performed with the program DICVOL.^14^ The whole powder pattern fitting (pattern-matching with the Le Bail fit) was done employing the FULLPROF program^15^ available in the software package WinPLOTR^16^ (Fig. S2†).

### 2.5. Thermal measurements

Thermogravimetric and differential thermal analyses were performed using a NETZSCH STA 449 instrument under nitrogen flux. The thermogram was collected on a 5 mg sample in the range of RT – 700 K, with a heating rate of 5 K min^−1^ in an aluminum capsule. Differential scanning calorimetry (DSC) analyses were carried out on raw powder with the NETZCSCH DSC 200 F3 instrument. The DSC measurement of [(CH_3_)_2_NH_2_]_2_PdBr_4_ was conducted over a temperature range from 300 K to 480 K with a heating and cooling rate of 5 K min^−1^. A sample of about 5 mg was placed in an Al_2_O_3_ crucible under nitrogen atmosphere.

### 2.6. UV-vis spectral measurements

The UV-vis absorption measurements were performed, in transmission configuration, at room temperature on a pellet with a diameter of approximately 8 mm and thickness of 0.4 mm using a standard UV-vis absorption spectrometer (Shimadzu, UV-3101PC) in the wavelength range of 200–1200 nm.

### 2.7. Raman measurements

Raman spectra were recorded between 60 cm^−1^ and 3000 cm^−1^ as a function of temperature using a Renishaw Invia Reflex spectrometer and a Linkam THMS600 temperature control stage. A He laser at 633 nm was used as the excitation source, with power of 1 mW. A X50 lens was used to focus and collect the incident and scattered lights, respectively. The scattered light from the sample was dispersed by a grating of 1800 lines per mm. At each temperature, the spectrum was acquired 10 minutes after the targeted temperature was achieved.

### 2.8. Electrical measurements

Electrical measurements were conducted on pellet disks with a thickness of 1 mm using the SOLARTRON SI 1260 impedance equipment coupled to a dielectric interface. The measurements were performed in the frequency range of 10^−1^–10^6^ Hz and the temperature intervals of 323–453 K with an AC voltage of 0.5 V. To ensure good contact, the pellet of 8 mm diameter and 1.2 mm thickness was linked with silver lacquer to the copper electrodes. The density of this material is equal to 4.39 g cm^−3^.

## 3. Results and discussion

### 3.1. Structural investigations

It is worth mentioning that the [(CH_3_)_2_NH_2_]_2_PdBr_4_ compound has already been reported in the literature.

Dimethylammonium cation was selected for this study because this organic cation is likely to exhibit orientational disorder, owing to its molecular flexibility and multiple points of interaction between organic and inorganic functionalities. The palladium(ii) bromide components contribute to semiconducting behavior and optical properties. In the same way, the reaction was focused on the study of the effects of using flexible organic moieties and Pd(ii) ion. Thus, the use of dimethylamine is one of the best ways to construct 2D hybrid halide perovskites with desirable physical properties. The crystal data (collected at 295 K) have been deposited in the Cambridge Crystallographic Database Centre, CCDC 1155080. This compound crystallizes in the orthorhombic crystal system (space group *Cmca*), with *a* = 18.457(4) Å, *b* = 7.320(2) Å, *c* = 9.844(2) Å, *V* = 1329.98 Å^3^ and *Z* = 4. However, the crystal structure of [(CH_3_)_2_NH_2_]_2_PdBr_4_ changes at 150 K into the monoclinic crystal system (space group *C*2/*c*) with the cell parameters *a* = 18.182(2) Å, *b* = 7.2741(7) Å, *c* = 9.8478(10) Å, *β* = 91.340(4)°, *V* = 1302.1(2) Å^3^ and *Z* = 4, as reported in the present work. The small changes in the unit cell parameters are attributed to the effect of temperature. At low temperature, the crystal structure is described as an organic–inorganic (2D) layered perovskite-derivative structure. The asymmetric unit (Fig. 1(b)) consists of one protonated dimethylammonium [(CH_3_)_2_NH_2_]^+^ cation and one half of a tetrabromopalladate(ii) [PdBr_4_]^2−^ anion. Fig. 1(c) shows a general view of the structure of [(CH_3_)_2_NH_2_]_2_PdBr_4_ along the *b*-direction, highlighting the molecular arrangement of organic cations in the interlayer space. The arrangement of the organic cation in the interlayer space is like that found in the structure of (CH_3_NH_3_)_2_PdX_4_ (X = Cl or Br),^17,18^ in which the methylammonium cations alternate with the inorganic anion layers to form a sandwich structure. For the inorganic part, Pd^2+^ is completed by sharing two bromine atoms from the adjacent PdBr_4_^2−^ ions along one axis. Consequently, the Pd^2+^ center is bridged by six bromine atoms, leading to corner-shared octahedral coordination geometry. In addition, within the inorganic layers, each [PdBr_6_]^4−^ anion displays Pd–Br bond lengths that vary from 2.4407(10) to 3.730(9) Å, see Table 2. The average bond lengths and bond angle values are like those found in other hybrid metal-halide perovskites.^18^ The degree of PdBr_6_ octahedron structural distortion was quantitatively evaluated by using the octahedron elongation Δ*d* parameter:^19^1
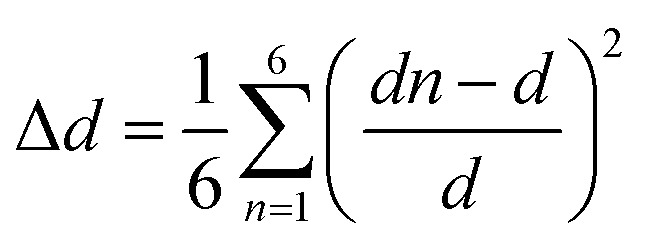
in which *d* is the average Pd–Br bond length and *dn* is the distance in each of the six individual Pd–Br bonds. The average Pd–Br distance is 2.873 Å, while Δ*d* is calculated to be 0.044, showing that the PdBr_6_ octahedron is highly distorted.

Moreover, as seen from the direction of the *a*-axis, adjacent PdBr_6_ octahedra are connected to the neighboring inorganic components through the bridging Br atoms, leading to a 2D anionic reticulated framework (Fig. 1(e)). Importantly, the infinite layers of corner-sharing PdBr_6_ octahedra are interleaved by the organic [(CH_3_)_2_NH_2_]^+^ cations, forming an alternating inorganic–organic architecture. In addition, the unique [(CH_3_)_2_NH_2_]^+^ cations reside in the cavities created by the inorganic layers. The main geometrical characteristics of the organic cation are listed in Table 2. The N–C bond lengths vary from 1.457(14) to 1.463(16) Å, and the value of the C–N–C angle is 115.9(10)°. These values are similar to those reported for comparable hybrid perovskites such as [(CH_3_)_2_NH_2_]PbX_3_ (X = Cl, Br),^20^ [(CH_3_)_2_NH_2_]PbI_3_ (ref. 21) and [NH_2_(CH_3_)_2_]_2_ZnBr_4_.^22^ As shown in Fig. 1(d), the inorganic layers connect the organic cations through intermolecular hydrogen bonds, with N–H⋯Br distances ranging from 3.341(9) to 3.489(9) Å and the bond angles ranging from 128 to 158°. Meanwhile, the C–H⋯Br interactions are in the range of 3.622(13)−3.989(12) Å, and the C–H⋯Br bond angles range between 122 to 149°. Detailed information on the hydrogen bonds is provided in Table 3. These hydrogen-bonding interactions can affect the optical and electrical properties of materials.^23^ Therefore, it is important to investigate the semiconductor properties of the 2D-layered hybrid perovskite materials.

### 3.2. Optical properties

**Fig. 2:**
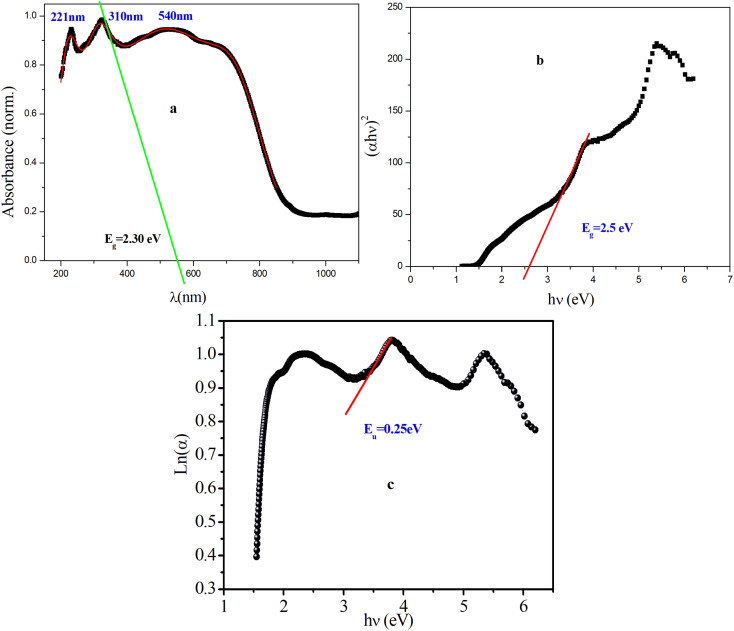
(a) Variation of the absorbance wavelength, (b) plot of (*αhν*)^2^*vs. hν*, (c) plot of ln(*α*) *vs. hν* for [(CH_3_)_2_NH_2_]_2_PdBr_4_.

The measurements of UV/vis absorption spectra were carried out in the range of 200–1200 nm, as shown in Fig. 2(a). As can be seen there, the peak at 310 nm (2.3 eV) with higher absorption in the UV range corresponds to the transition of an electron from the valence band to the conduction band in the tetrahedron PdBr_4_^2−^. This peak is due mainly to the absorption between Br (5p) and Pd (4d) (band to band), which suggests that the material behaves as a semiconductor.^24^ The other peaks observed around 221 nm and 540 nm can be due to a photoinduced exciton formed in the inorganic complexes^25^ and the orange color of the sample, respectively.

According to the Tauc equation,^26^ the corresponding optical band gap can be defined as 2.5 eV for this compound (Fig. 2b), which indicates that [(CH_3_)_2_NH_2_]_2_PdBr_4_ is a semiconductor and a potential optoelectronic candidate. This result is confirmed by extrapolation of the absorption band at 310 nm (Fig. 2a). Compared to similar compounds with 2D structure, such as [(CH_3_)_2_NH_2_]_2_PbBr_3_, [(CH_3_)_2_NH_2_]_2_PbCl_3_ and [(CH_3_)_2_NH_2_]_2_ZnBr_4_, where the gap energies are 3 eV, 3.5 eV and 4.76 eV, respectively, [(CH_3_)_2_NH_2_]_2_PdBr_4_ presents the lowest band gap.^26,29^ The difference in gap energy among these compounds can be attributed to the change of metal ion. In addition, to further investigate the pre-absorption edge and obtain information on the disorder in a material, an Urbach tail analysis was performed, as presented in Fig. 2c. The Urbach energy value, *E*_u_, is estimated to be 0.25 eV, which is lower than the values of similar compounds such as [(NCH_3_)_2_H_2_]_2_CoCl_4_, [(NCH_3_)_2_H_2_]_2_ZnCl_4_ and [(NCH_3_)_2_H_2_]_2_ZnBr_4_, indicating that this compound is less disordered.^27,28^

### 3.3. Thermal behavior

**Fig. 3:**
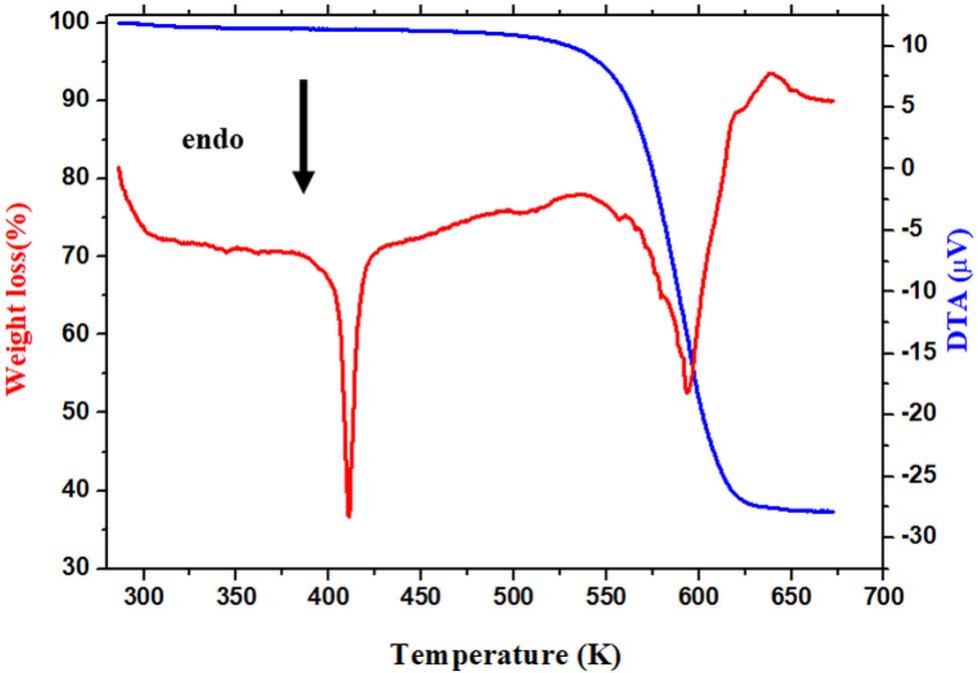
DTA and TGA curves for the [(CH_3_)_2_NH_2_]_2_PdBr_4_ crystal during the heating run.

**Fig. 4:**
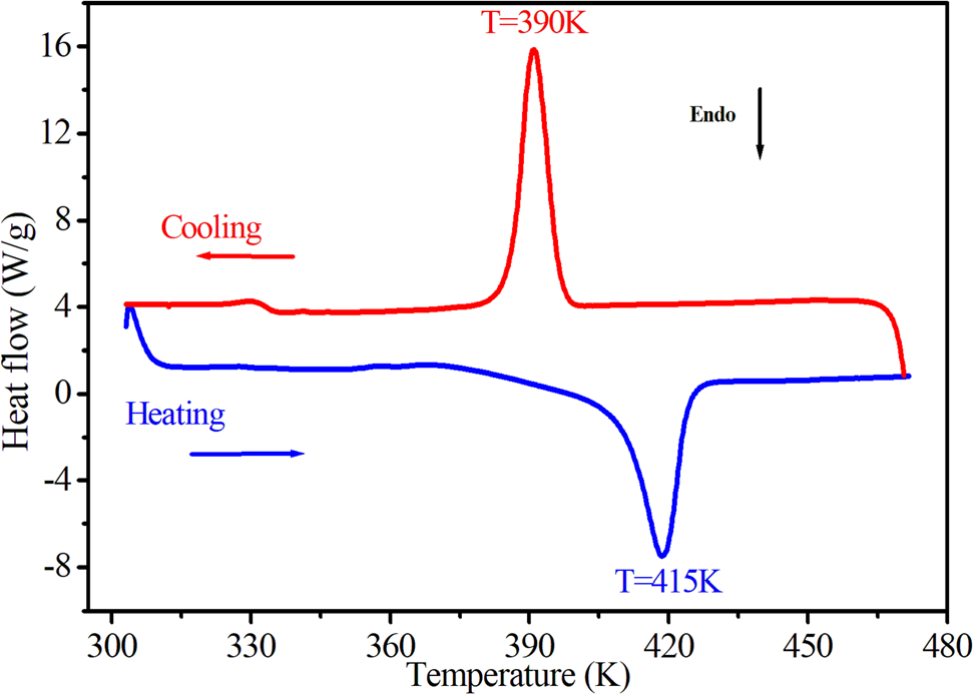
DSC results as a function of temperature, obtained by heating and cooling the [(CH_3_)_2_NH_2_]_2_PdBr_4_ compound at a rate of 5 K min^−1^.

The thermal behavior of [(CH_3_)_2_NH_2_]_2_PdBr_4_ was studied using thermogravimetry, differential thermal analysis (TGA-DTA, see Fig. 3), and differential scanning calorimetry (DSC, see Fig. 4). The DTA experiment displays an endothermic peak on heating at *T* ∼ 415 K, which is associated to an exothermic peak appearing on cooling at slightly lower temperature (at *T* ∼ 390 K) and which can be attributed to a melting followed by rapid decomposition of the organic part (confirmed later by Raman spectroscopy studies as a function of temperature).

As seen in Fig. 3, the measured DTA dependence shows a clear anomaly at about 415 K. According to the TGA results, thermal stability of the compound is seen well up to about 500 K. The hybrid material decomposes in one step, with a weight loss of about 51% corresponding to the decomposition of the two organic parts, and about 48.62% attributed to the two bromine atoms. This phenomenon is usually observed in the field of hybrid halide perovskites.^19,29^ Therefore, the observed anomalies at 415 K in both the DSC and DTA measurements provide evidence for the occurrence of melting in this material, and the thermal stability of extends up to approximately 500 K.

### 3.4. Raman spectroscopy

**Fig. 5:**
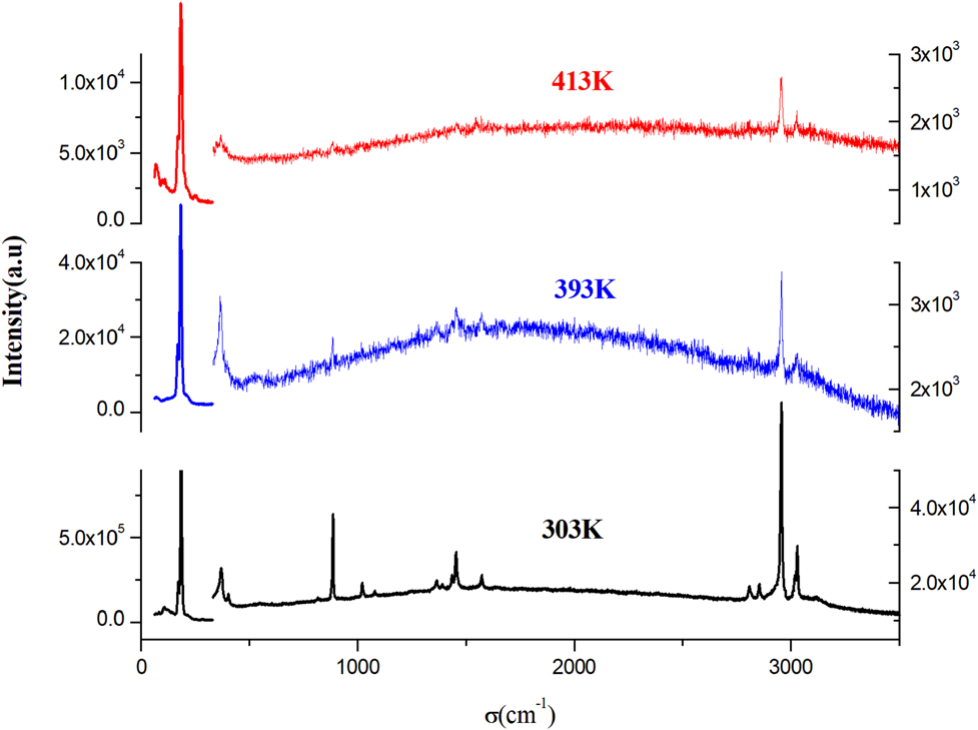
Raman spectra of [(CH_3_)_2_NH_2_]_2_PdBr_4_ at various temperatures.

**Fig. 6:**
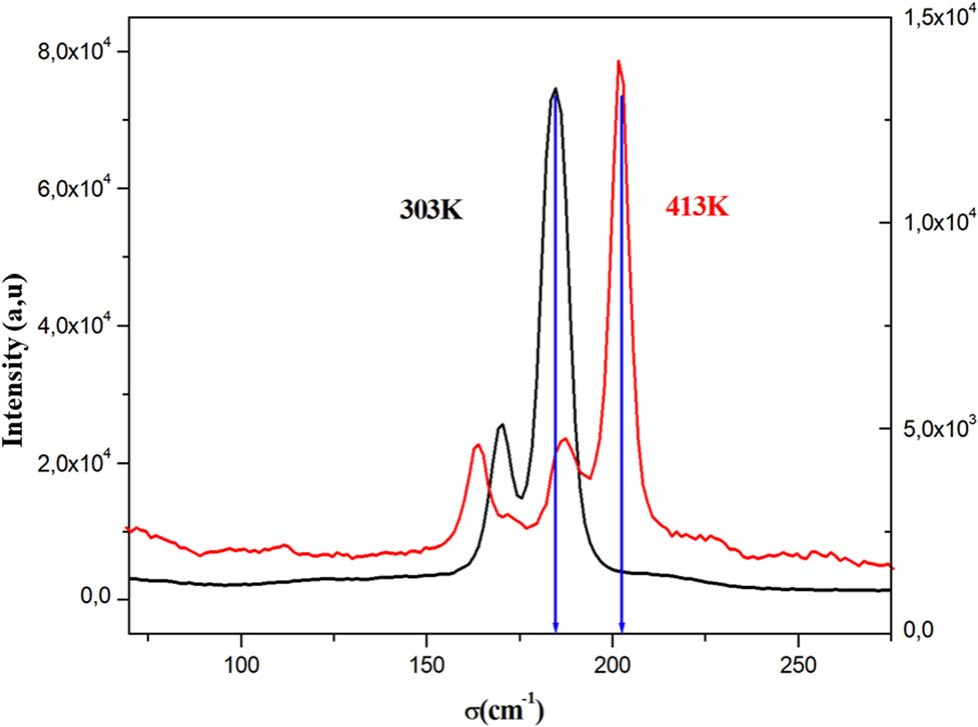
The position of PdBr_4_^2−^ vibration modes before and after the melting temperature of [(CH_3_)_2_NH_2_]_2_PdBr_4_.

To identify the presence of the different entities (organic cation and inorganic anion) and to determine the nature of the peak observed in the DSC, we recorded Raman spectra as a function of temperature (Fig. 5). The Raman spectrum at 303 K shows three regions: the first, at low frequency and below 350 cm^−1^, corresponds to the vibration modes of the inorganic anion;^30^ the second, between 350 and 1600 cm^−1^, to the stretching mode of the N–C vibrations and vibrational modes of the CH_3_ groups; the last region at higher frequencies corresponds to the elongation vibration of C–H and N–H vibration modes.^31^ This analysis confirms the presence of all vibration modes of the two organic and inorganic entities. At 393 K, near the observed endothermic peak in the DSC thermogram, the recorded Raman spectrum shows the melting of the organic part of this material. Indeed, the peaks above 350 cm^−1^ begin to disappear; this result is confirmed by the spectrum at 413 K, where most peaks of the organic entity completely disappear, which allows us to attribute the anomaly observed in the DSC to melting of the organic entity of this material. Fig. 6 shows the position of PdBr_4_^2−^ vibration modes before and after the melting temperature. We notice the change in the position of the inorganic entity, which is due to the breaking of N–H⋯Br bonds resulting from the melting of the organic cation and the change of the cell volume of this material after melting.

### 3.5. Impedance spectroscopy analysis

**Fig. 7:**
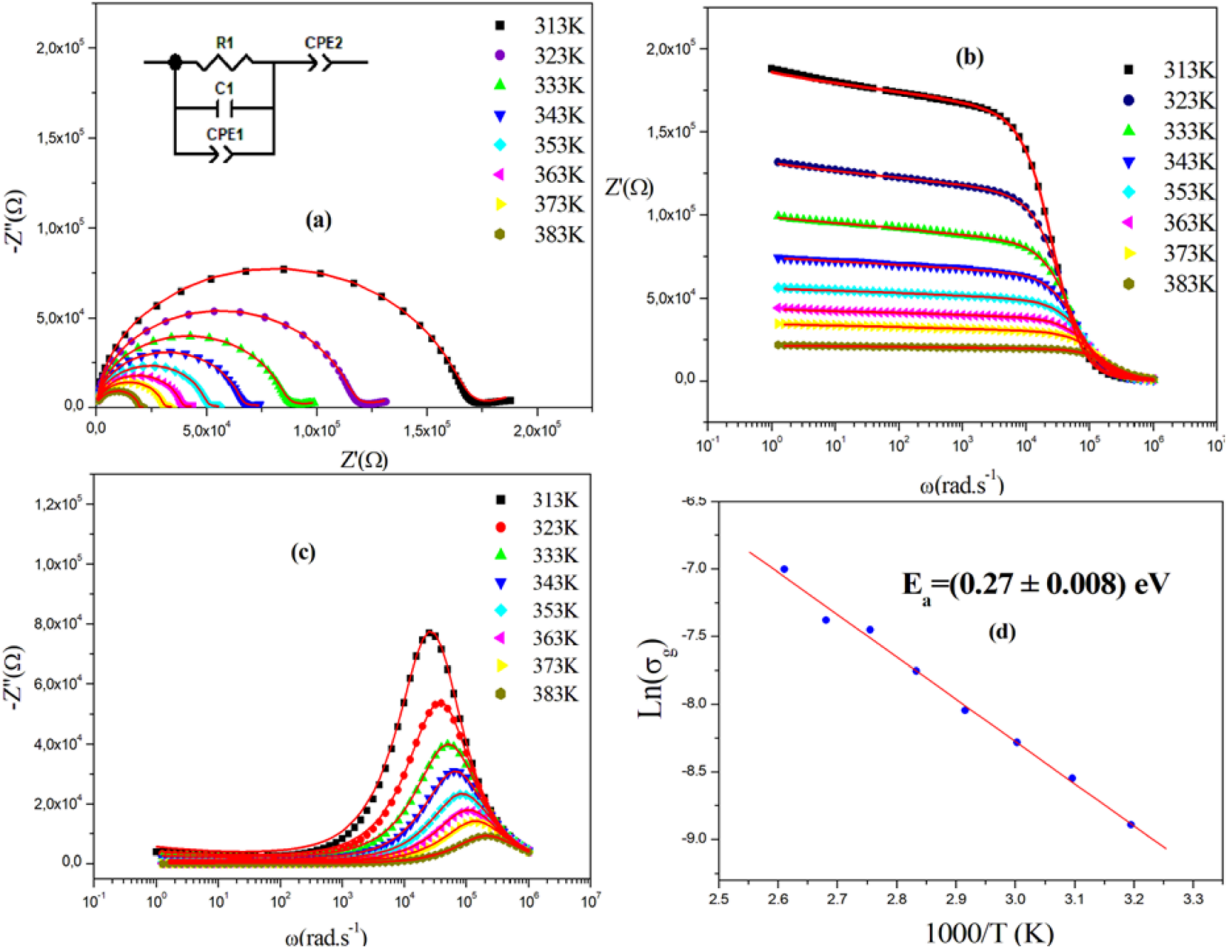
(a) Nyquist plots and equivalent circuit model of [(CH_3_)_2_NH_2_]_2_PdBr_4_. (b) Variation of the imaginary part and (c) real part of impedance as a function of frequency, and (d) the plot of ln(*σ*_g_) *versus* 1000/*T*.

Fig. 7(a) displays the complex impedance spectra of (C_2_H_8_N)_2_PdBr_4_ at various temperatures, shown as *Z*′′ against *Z*′. Instead of a semicircle centered on the real axis, all impedance spectra show some dispersion, which suggests a non-Debye type of relaxation.^32,33^ The best fit using Z-view software was obtained using an equivalent circuit formed by a parallel combination of bulk resistance *R*_g_, real capacity *C*_g_, fractal capacity CPE_g_ and free CPE. The two parallel capacity values, *C* and CPE, indicate the overlapping between the grain and the grain boundary response. The diameters of the semicircles decrease with increasing temperature, indicating a thermally activated conduction mechanism. This is consistent with the associated grain resistance value (*Z*′) (Fig. 7b) and the progressive decrease of maximum 
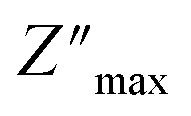
 (Fig. 7c) as the temperature increases. The 
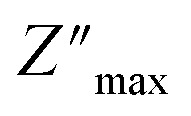
 rises and shifts to high frequencies, indicating the accumulation of space charge in the material and confirming the occurrence of non-Debye relaxation.^34,35^ Compared to a similar compound reported by A. Ben Rhaiem *et al.*,^36^, [(CH_3_)_2_NH_2_]_2_PdBr_4_ shows a 
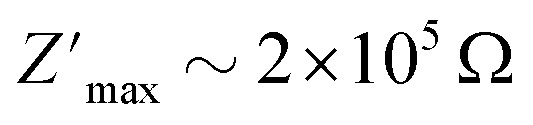
, which is lower than the 
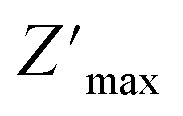
 value of [(CH_3_)_2_NH_2_]_2_ZnBr_4_ ∼1.6 × 10^6^ Ω.

The obtained values of bulk resistance (*R*_g_), corresponding to the grain, are used to determine the electrical conductivity *σ*_g_ as follows:^37^2
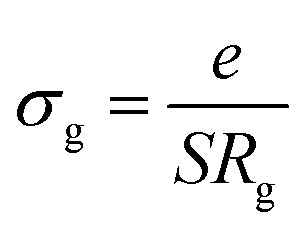
where *e* is the thickness of the sample and *S* is the area of one flat face of the pellet. The thermal dependence of *R*_g_ and the electrical conductivity ln(*σ*_g_) *versus* (1000/*T*) is displayed in Fig. 7d. The conductivity is strongly temperature dependent and follows Arrhenius law:3
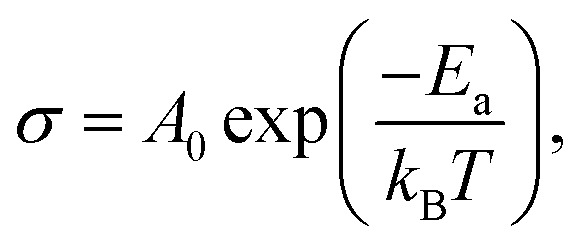
where *E*_a_ is the activation energy. The estimated activation energy is 0.27 ± 0.008 eV, which is close to that of ionic-type conduction.^38^ The optical band gap displayed by this material *E*_g_ = 2.5 eV, and the value of activation energy rules out the possibility that the observed conductivity can be electronic and instead points to ionic conductivity.^21^

### 3.6. Complex modulus analysis

**Fig. 8:**
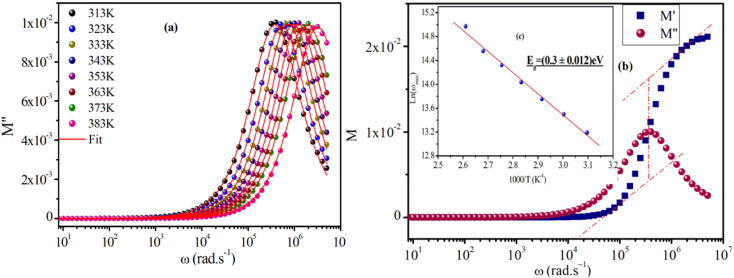
(a) Angular frequency dependence of the imaginary part of the electric modulus at several temperatures for the [(CH_3_)_2_NH_2_]_2_PdBr_4_ compound, (b) using the tangent method of *M*′; (inset, c) the plot of ln(*ω*_gmax_) *versus* 1000/*T*.

Fig. 8a represents the frequency dependence of the imaginary part (*M*′′) of the modulus at different temperatures. The peak maximum 
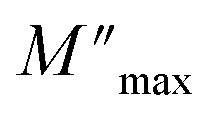
 shifts to higher frequencies with increasing temperature, indicating that the dielectric relaxation process is thermally activated, where the hopping process of charge carriers overcomes intrinsic barriers.^39,40^ This behavior confirms the non-Debye-type relaxation (single relaxation time).^41^ The imaginary part of the electric modulus at different temperatures has been fitted with the function proposed by Bergman:^42^4
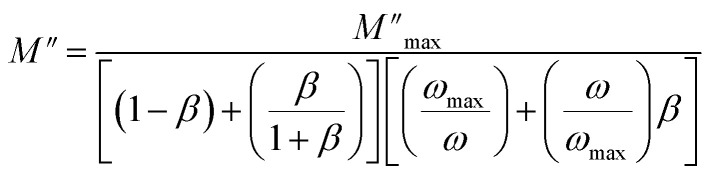


At 
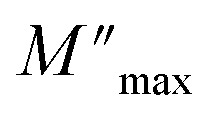
, the maximum and minimum tangent points coincide (Fig. 8b), which agrees well with Bergman's fit. Additionally, based on Bergman's fit, the variation of *ω*_max_ of 
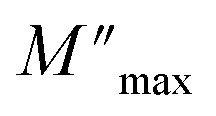
 shows an activation energy for intrinsic conduction equal to (0.3 ± 0.012) eV (Fig. 8c).

**Fig. 9:**
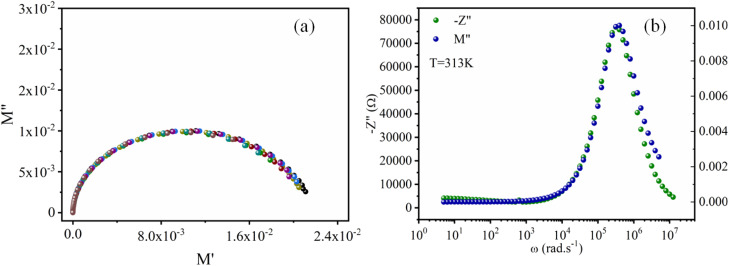
(a) Plots of the imaginary modulus *M*′′ *vs.* real modulus *M*′. (b) Frequency dependence of relaxation peaks, *M*′′ and −*Z*′′, for [(CH_3_)_2_NH_2_]_2_PdBr_4_.

Fig. 9a displays the complex modulus spectrum (*M*′′ *versus M*′) of [(CH_3_)_2_NH_2_]_2_PdBr_4_ at selected temperatures. It shows a single semicircular arc, where the intercept with the real axis indicates the capacitive contribution of the grains. It also confirms that the effect of bulk boundaries is negligible. The non-semicircular shape of *M*′′ *versus M*′ confirms the occurrence of non-Debye-type relaxation.^43^ The arcs were perfectly overlapped into a single master curve, indicating that the underlying conduction mechanism remains the same. Furthermore, Fig. 9b shows that the peak positions of *M*′′ and *Z*′′ overlap, indicating that long-range and localized relaxations occur simultaneously. This superimposition is characteristic of a long-range electronic conductivity within grains,^44^ which is responsible for the observed high-temperature dielectric relaxation.^45,46^

### 3.7. Frequency dependence of capacitance and conductivity

**Fig. 10:**
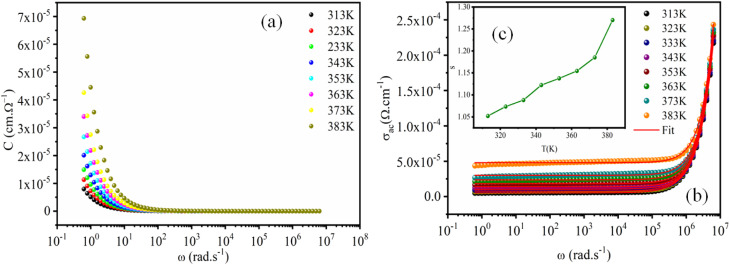
Frequency dependence of the capacitance (a) and AC conductivity (b) at various temperatures for the [(CH_3_)_2_NH_2_]_2_PdBr_4_ compound. (inset, c) The variation of universal exponent *s* as a function of temperature.

To identify the mechanism of conduction, capacitance measurements were performed at the same frequencies and temperatures. Fig. 10a shows the capacitance curve C (frequency) as a function of temperature for the perovskite (C_2_H_8_N)_2_PdBr_4_. At lower frequencies (*f* < 10^2^ Hz), the capacitance is strongly dependent on temperature, which is attributed to ion and charge accumulation near the contact interfaces or to electrode polarization.^47^ However, at high frequencies (*f* > 10^2^ Hz), the capacitance becomes saturated, reaching a plateau. This is due to the change in space charge, ionic and orientation polarization at higher frequencies.

On other hand, the variation of AC conductivity *σ*_AC_ (Fig. 10b) as a function of frequency and temperature showed values in the order of 10^−4^ Ω^−1^ cm^−1^, which confirms the semiconductor character of this material and the optical results. These spectra obey the Jonscher power law given by the following equation:

5 *σ*_AC_ = *σ*_DC_ + *Aω*^*s*^,

where *σ*_DC_ is the conductivity at low frequencies, *A* is a parameter dependent on temperature, and *s* is the power law exponent with values in the range 1 < *s* < 2.^48^

At low frequencies, the conductivity has a plateau (*σ*_DC_) defined by the first term of the Jonscher equation; when the temperature increases, the bearing becomes increasingly clearer, which indicates that this regime is activated thermally. At high frequency, the conductivity exhibits an asymptotic form (or dispersive regime), which can be defined by the second term *Aω*^*s*^, where the charge species accumulated at the grain boundaries have sufficient energy to overcome the energy barrier upon increasing temperature. The spectra of the conductivity can be fitted by the eqn (5) to extract the exponent *s* at various temperatures in order to determine the conduction mechanisms.

The reduced gap energy (2.5 eV) and high conductivity, in the order of 10^−4^ Ω^−1^ cm^−1^, proves that this material is a semiconductor that can be used in photovoltaic cells in the role of a photodetector that converts the optical signal into an electrical signal. The properties qualifying this material as an optoelectronic material is the thermal stability, which reaches up to 390 K due to the use of palladium.

### 3.8. Electrical conduction mechanisms

Fig. 10c (inset) displays the temperature dependence of the *s* frequency exponent. It can be noted that the exponent *s* increases with the temperature, indicating that the prevailing model is not overlapping small polaron tunneling (NSPT).^49^

AC conductivity depends on some parameters, such as the density of states at Fermi's level N(*E*_F_), the spatial extension of the polaron *α*, and the jump energy of polaron *W*_H_ (eqn (6)).^50^ These parameters could be determined from the fit of the experimental data as shown in Fig. 8.6
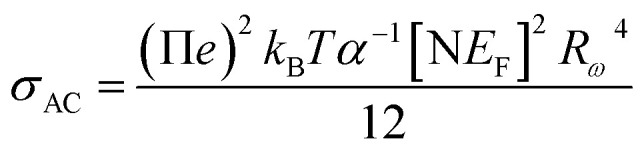


with corresponding jump distance7
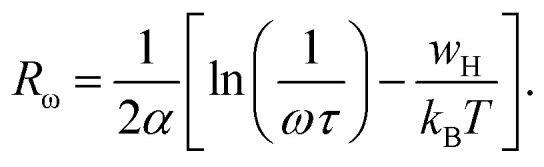


**Fig. 11:**
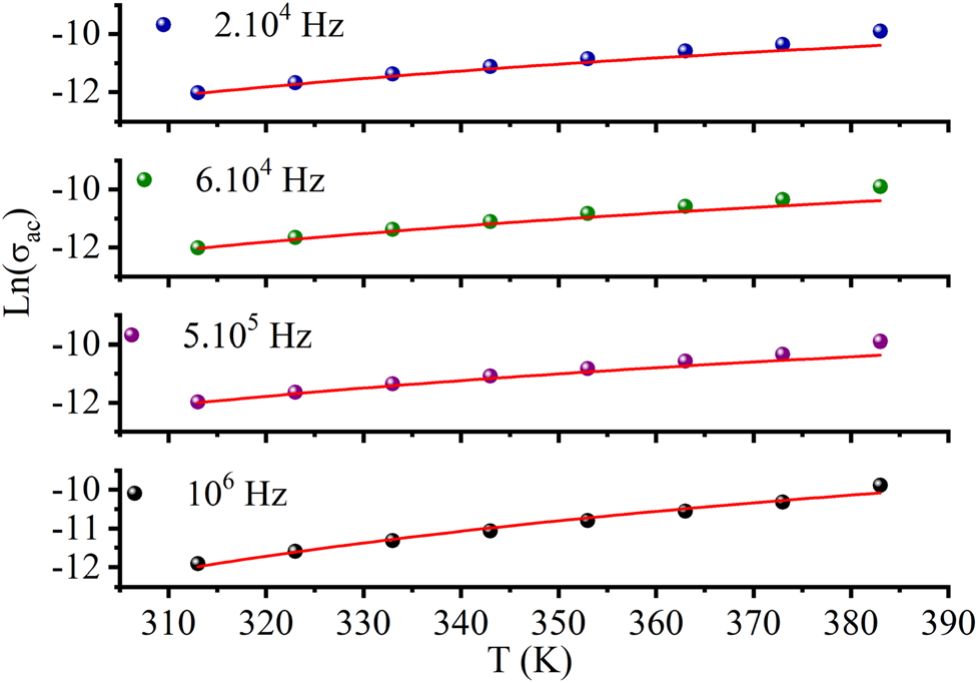
Fitting of AC conductivity at different frequencies using the NSPT model.

The parameters obtained from the fit of the curve (Fig. 11) are listed in Table 4. The small variation in the jump energy of polaron *W*_H_ confirms the polaron located in the quantum “well” between two inorganic layers. The spatial extension of the polaron *α*^−1^ increases with decreasing frequency, suggesting that the hopping of charges is carried out between the nearest-neighbor sites.

**Table 4:** AC conductivity parameters N(*E*_F_) (eV^−1^ m^−3^), *α* (Å ^−1^) and *ω*_H_ (eV) in [(CH_3_)_2_NH_2_]_2_PdBr_4_ at various frequencies

Frequency (Hz)	N(*E*_F_) (eV^−1^ m^−3^)	*α* (Å^−1^)	*ω*_H_ (eV)	*R* (Å)
10^6^	6.66 × 10^19^	1.0438	0.4166	0.42
5 × 10^5^	6.75 × 10^19^	1.0380	0.4206	0.45
6 × 10^4^	6.35 × 10^19^	1.0646	0.4111	1.58
2 × 10^4^	6.34 × 10^19^	2.0648	0.4115	1.08

## 4. Conclusion

[(CH_3_)_2_NH_2_]_2_PdBr_4_ has been synthesized, and its crystal structure determined from single-crystal X-ray diffraction data. The compound crystalizes in the monoclinic system (*C*2/*c*), and its structure consists of a 2D perovskite-like structure with alternating organic and inorganic layers. The 2D halide perovskite [(CH_3_)_2_NH_2_]_2_PdBr_4_ exhibits good optical properties, with a direct band gap of *E*_g_ = 2.5 eV. This result is confirmed by the AC conductivity, which shows values in the order of 10^−4^ Ω^−1^ cm^−1^. The thermal properties indicate the stability of this material up to 500 K, and Raman spectra show the presence of both organic and inorganic entities and confirm the melting of the organic molecule at 413 K. The activation energy has been determined, and the conduction mechanisms are identified in this material. [(CH_3_)_2_NH_2_]_2_PdBr_4_ is a promising candidate for optical applications such as laser or solar cells.

## Conflicts of interest

There are no conflicts to declare.

## Supplementary Material

RA-013-D3RA04085B-s001

RA-013-D3RA04085B-s002
