# Supplementary material for: [(CH3)2NH2]2PdBr4, a layered hybrid halide perovskite semiconductor with improved optical and electrical properties
Source: RSC Adv. 2023 Aug 3;13(33):23348–58. doi: 10.1039/d3ra04085b (PMC10399296; doi:10.1039/d3ra04085b)
Supplement: RA-013-D3RA04085B-s001 [file RA-013-D3RA04085B-s001.pdf]

Electronic Supplementary Material (ESI) for RSC Advances.
This journal is © The Royal Society of Chemistry 2023

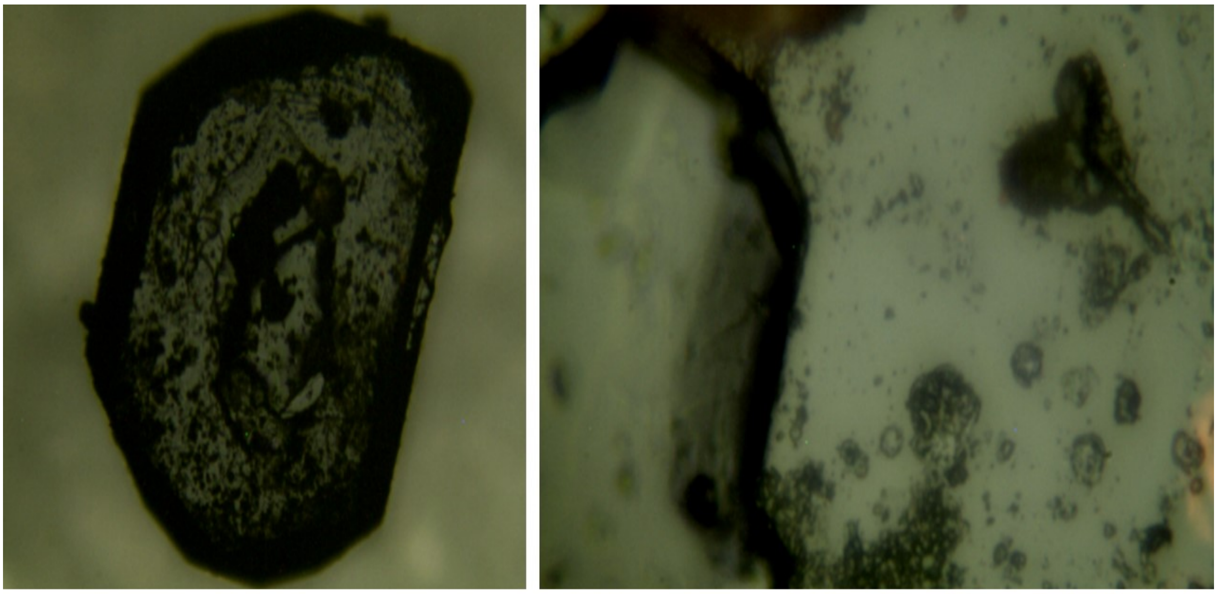

a **b**

Figure. S1: (a) Micrograph shows the dimensions of crystal for $[(CH_3)_2NH_2]_2PdBr_4$

(b) the image of the surface of the crystal taken with the objective ×50 of a microscope.

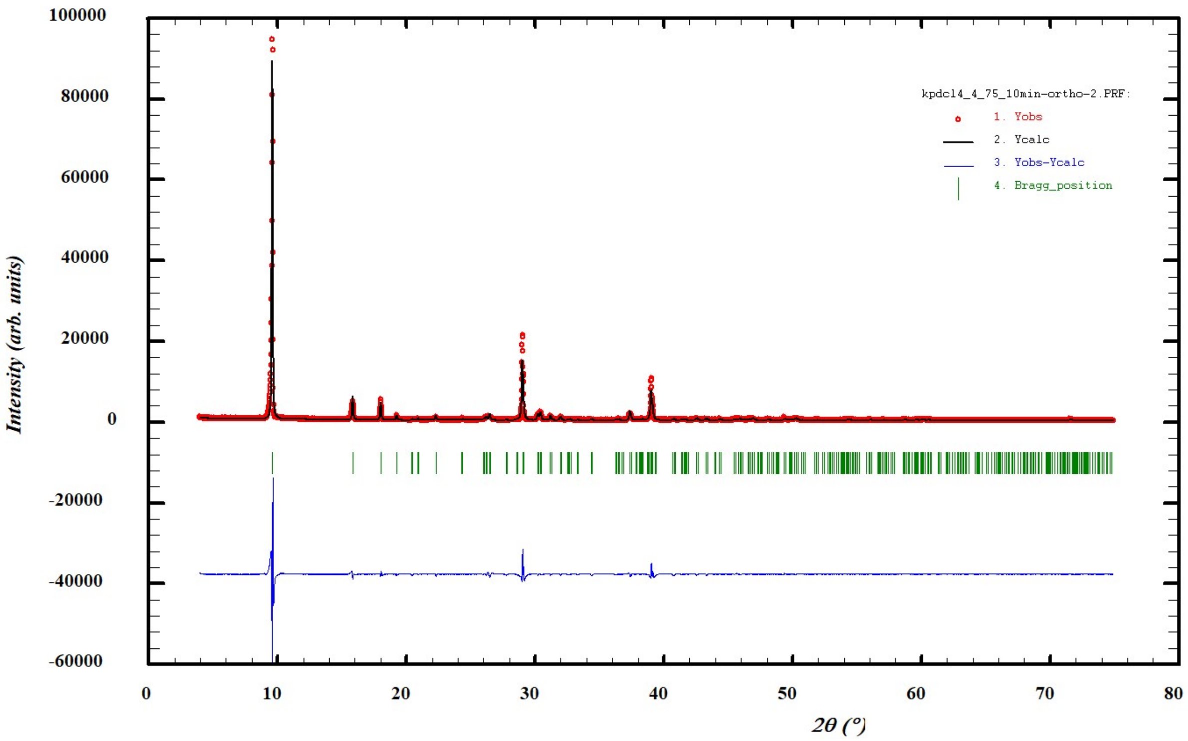

Figure. S2: Plot of the Bail fit of the powder X-ray diffraction pattern recorded at 293 K for $[(CH_3)_2NH_2]_2PdBr_4$: *Cmca*, *a* = 18.4521(8) Å, *b* = 7.3181(8) Å, *c* = 9.8482(9) Å; Rp = 13.9, Rwp = 21.8, Rexp = 3.53.
